# Insect egg-induced innate immunity: Who benefits?

**DOI:** 10.1371/journal.ppat.1011072

**Published:** 2023-01-19

**Authors:** Elia Stahl, Louis-Philippe Maier, Philippe Reymond

**Affiliations:** Department of Plant Molecular Biology, University of Lausanne, Lausanne, Switzerland; Shanghai Center for Plant Stress Biology, CHINA

## Abstract

Plants perceive the presence of insect eggs deposited on leaves as a cue of imminent herbivore attack. Consequential plant signaling events include the accumulation of salicylic acid and reactive oxygen species, transcriptional reprogramming, and cell death. Interestingly, egg-induced innate immunity shows similarities with immune responses triggered upon recognition of microbial pathogens, and in recent years, it became apparent that egg perception affects plant–microbe interactions. Here, we highlight recent findings on insect egg-induced innate immunity and how egg-mediated signaling impacts plant–microbe interactions. Ecological considerations beg the question: Who benefits from egg perception in these complex interactions?

## Perception of insect eggs

Throughout their life cycle, plants interact with a multitude of biotic stressors and have therefore evolved an elaborated immune system to counteract such threats. Initiation of plant immune signaling involves recognition of conserved molecular patterns of the aggressor by plasma membrane-localized pattern recognition receptors, a process called pattern triggered immunity (PTI). Similarly, activation of insect egg-induced immune signaling depends on the recognition of specific egg-associated molecular patterns (EAMPs) and not on microbial patterns associated with insect eggs [1,2]. Initiation of plant immune signaling upon egg recognition was reported for insects from different orders, including butterflies and moths (Lepidoptera), planthoppers (Hemiptera), bugs (Hemiptera and Heteroptera), beetles (Coloptera), and sawflies (Hymenoptera) [1]. Known EAMPs include small molecules, such as indole, benzyl cyanide, bruchins, and phosphatidylcholines (PCs), as well as the annexin-like protein diprionin [3,4]. Although several EAMPs and their physiological effects on plants have been described, their cognate receptors remain to be identified and no direct receptor–ligand interaction has been demonstrated so far. However, in the model plant *Arabidopsis thaliana* (hereafter *Arabidopsis*), the L-TYPE LECTIN RECEPTOR KINASE I.8 (LecRK-I.8) has been reported as a crucial component of egg perception from different insect species, such as the large white butterfly *Pieris brassicae*, the Egyptian cotton worm *Spodoptera littoralis*, and the cabbage looper *Trichoplusia ni* [5–7]. Induction of PTI in response to egg-derived PCs depends partially on LecRK-I.8, suggesting a role for this putative receptor in PC-induced immune signaling [8]. Intriguingly, the same receptor has been described as a sensor for extracellular NAD^+^ [6]. However, a relation of NAD^+^-mediated signaling in insect egg-induced immunity has not been described yet and will be an interesting topic for further investigations. A genome-wide association study has recently discovered that the L-TYPE LECTIN RECEPTOR KINASE I.1 (LecRK-I.1), a close paralog of LecRK-I.8, is involved in egg-mediated cell death in *Arabidopsis* [9]. Additionally, an ortholog of LecRK-I.1 may potentially regulate egg-induced cell death in the Chinese cabbage *Brassica rapa* [10]. Together, these data indicate that responsiveness to insect eggs is controlled by multiple LecRKs in cruciferous plants.

**Fig 1:**
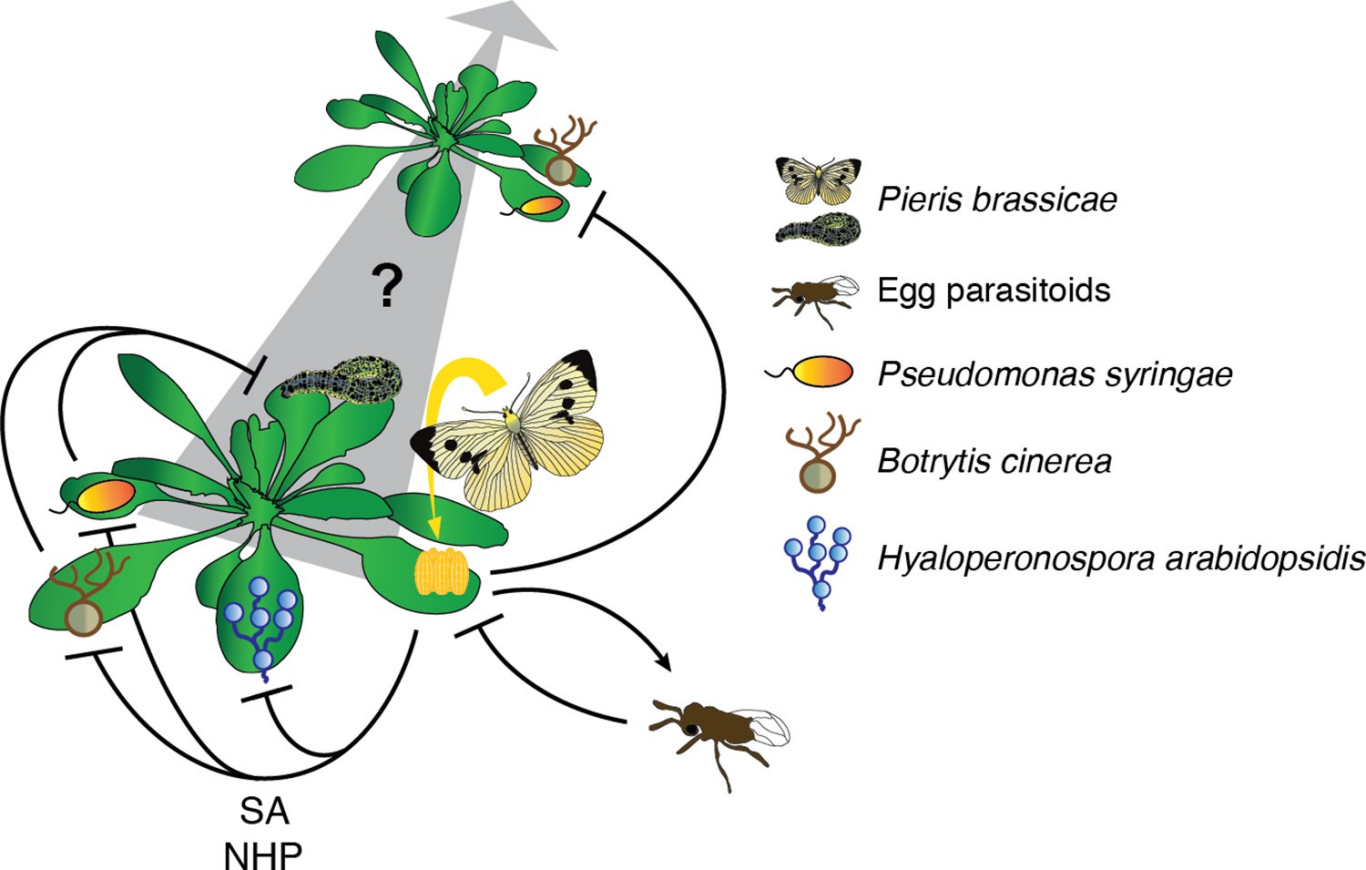
Insect egg-triggered immunity affects plant–microbe interactions in *Arabidopsis*. *Arabidopsis* plants perceive egg deposition and respond with a local induction of innate immunity. Emission of leaf volatiles leads to the attraction of egg parasitoids, which impedes egg survival. Activated signaling events lead to an increased resistance in the full plant foliage against microbial pathogens with different lifestyles including the bacterial plant pathogen *Pseudomonas syringae*, the fungal plant pathogen *Botrytis cinerea*, and the oomycete pathogen *Hyaloperonospora arabidopsidis*. Induction of this egg-induced SAR fully depends on SA and NHP signals and, surprisingly, also takes place in neighboring plants via yet unknown root-derived signal(s) (grey arrow). Interestingly, larvae of the specialist herbivore *Pieris brassicae* perform less well when feeding on plants infected with *P*. *syringae* and *B*. *cinerea*, indicating that activation of SAR in response to oviposition could be a strategy to ensure a healthy host plant to feed hatching larvae. NHP, N-hydroxypipecolic acid; SA, salicylic acid; SAR, systemic acquired resistance.

Perception of EAMPs induces typical immune responses in *Arabidopsis*, including the accumulation of reactive oxygen species (ROS), immune regulatory signals salicylic acid (SA) and pipecolic acid (Pip), and indolic metabolites [4,11,12]. Moreover, egg recognition triggers extensive transcriptional reprogramming with an up-regulation of immunoregulatory and defense-related genes at the expense of photosynthesis and development [13–15]. Hypersensitive response-like (HR-like) lesions develop on leaf tissue underneath the eggs. This programmed cell death is SA dependent, requires sphingolipid metabolism, and impedes egg survival [1,16]. Interestingly, the strength of HR-like in the crucifer family depends on the plant and insect species considered. A particularly strong HR-like is observed in a clade including several crops, such as cabbage (*Brassica oleracea*) and close relatives. In addition, this response is more pronounced in response to eggs from crucifer-specialized pierids, which have the capacity to detoxify glucosinolates, the main defense compounds from in this plant family. These observations suggest that egg killing by HR-like cell death is a defense trait which cruciferous plants have evolved against specialized herbivore species [17]. Although HR-like necrosis is a local phenomenon that is limited to the site of egg deposition, the activation of other immune responses is not limited to the perception site. Accumulation of SA, Pip, specific indoles, and *PR1* transcript levels were also measured in leaves distal to the site of initial egg recognition [12,18], indicating an activation of systemic immunity upon local egg perception. In addition to direct immune responses, plants also employ indirect defense responses against insect eggs. For instance, plants emit a bouquet of volatile compounds in response to oviposition that attract egg parasitoids, such as the wasp *Trichogramma brassicae*, whose offspring in turn kills the herbivore eggs (Fig 1) [1,4]. However, information on the signaling pathways leading to volatile emission upon oviposition is scarce and an interesting aspect for future studies.

## Insect eggs trigger plant resistance against microbial pathogens

Local pathogen exposure and activation of immunity lead to an increased broad-spectrum resistance against microbial pathogens in the whole plant foliage, a complex phenomenon called systemic acquired resistance (SAR) [19,20]. Strikingly, egg-induced PTI also increases plant resistance against different strains of the hemibiotrophic bacterial pathogen *Pseudomonas syringae* (*Pst*), the necrotrophic fungal pathogen *Botrytis cinerea*, and the biotrophic oomycete pathogen *Hyaloperonospora arabidopsidis*, both at the site of oviposition and in distal leaves, indicating that egg perception triggers SAR (Fig 1) [12,18]. In *Arabidopsis*, microbial-induced SAR is (1) regulated by N-hydroxypipecolic acid (NHP) and SA signals [21]; (2) tightly associated with priming of defenses, a status in which a plant responds more quickly and vigorously to a subsequent pathogen infection [22,23]; and (3) accompanied by an activation of indolic metabolism [24]. Egg-induced SAR displays the same characteristics, demonstrating that oviposition triggers a SAR that is highly similar to that induced by microbial pathogens [12,18].

Intriguingly, egg-induced SAR in *Arabidopsis* is not limited to the oviposited plant. Indeed, plants growing next to egg-laden neighbors show increased resistance against *Pst* and *B*. *cinerea* (Fig 1) [12,25]. Like the intraplant SAR, this interplant SAR depends on functional NHP and SA signaling pathways. Moreover, egg-treated plants produce a belowground signal to trigger defenses in receiving plants [24]. However, the chemical nature of the root-derived compound is not known and remains to be elucidated. Although it was shown that distinct immune responses, such as the accumulation of SA, are induced in plants neighboring egg-laden plants, the full extent of immune activation has not been investigated yet. For instance, whether emission of parasitoid-attracting volatiles is triggered in receiver plants is an intriguing hypothesis that will deserve further investigation.

## Who benefits?

The ecological role of insect egg-induced intra- and interplant SAR is an intriguing, yet not fully resolved, question. The activation of SA in response to egg recognition was initially investigated for its impact on plant defense against chewing larvae. Indeed, it is well established that increased SA levels repress jasmonic acid (JA)-mediated signaling, which mainly orchestrates plant immunity against chewing herbivores [26,27]. Accordingly, larvae of the generalist *S*. *littoralis* performed better on *Arabidopsis* when plants were previously exposed to *S*. *littoralis* or *P*. *brassicae* eggs, and this effect was dependent on functional SA biosynthesis [28]. This finding suggests that generalist herbivorous insects may benefit from activating the SA pathway, although this could be at the cost of reduced egg survival. The potential fitness cost for plants incurred by enhancing future generalist larval performance through SA/JA crosstalk may be less pricey compared to the opportunity to decrease the total imminent herbivore load by impeding egg development. In contrast, biomass gain of larvae of the specialist *P*. *brassicae* was not affected, or decreased, in response to *P*. *brassicae* eggs, indicating that that the outcome of egg-induced signaling on larval fitness may depend on the insect species considered [28,29]. Moreover, SA and NHP signaling pathways have been previously reported to be involved in the regulation of stress-inducible emission of volatiles in *Arabidopsis* and treatment with SA leads to the emission of a bouquet of volatiles in tomato [30–32]. Therefore, activation of SA and NHP signaling pathways might be additionally involved in regulating oviposition-induced volatile emission, which constitutes a crucial indirect plant defense against insect eggs.

Alternatively, as wounding occurs during herbivory, activation of SA signaling and SAR in response to eggs may protect plants against potential infections from opportunistic pathogens. Bacterial plant pathogens, such as *Pst*, enter host leaves through natural openings or wounds [33,34]. Moreover, the microbial community of the phyllosphere is modulated by herbivory, and the bacterial load of pathogenic bacteria, such as *P*. *syringae*, increases [35]. Thus, egg-induced immunity may be the evolutionary outcome of a trade-off between enhancing larval performance through SA/JA crosstalk, impeding egg development via an HR-like, and reducing the threat of wound-related infection via SAR. However, an increase in pathogen load may be detrimental not only to the plant but also to the attacking herbivore. *P*. *brassicae* larvae grow slower when feeding on *Arabidopsis* plants infected with *Pst* or *B*. *cinerea* (Fig 1) [12,18], pointing to the additional hypothesis that, from an insect-centric point of view, egg-induced SAR creates a healthy and nutrient-rich plant environment for feeding larvae. This implies that, by releasing egg-derived EAMPs, insects may have evolved a strategy to hijack the SA pathway to protect host plants against microbial pathogens and therefore to benefit survival of their progeny. This idea blurs the boundaries surrounding the concept of EAMPs/PAMPs being only elicitors of plant defenses and adds a putative function as defense suppressing molecules. However, since they are not mutually exclusive, these hypotheses illustrate a situation in where both insects and plants may profit from activation of the same signaling pathway. It is well established that SA/JA crosstalk is exploited by microbial pathogens to support their virulence. Indeed, necrotrophic pathogens evolved mechanisms to modulate SA signaling to suppress JA-mediated immunity, whereupon biotrophs hijack the JA pathway for suppression of the SA pathway [36]. It will be interesting for future studies to investigate how microbe-mediated modulation of defense signaling pathways affects insect herbivore performance and if microbes benefit from such processes in a natural setting, where microbial plant colonization and herbivore attack happen simultaneously.

The role of egg-induced interplant SAR is an even more complex question. Although the release of volatile or belowground signals by plants and their perception by neighbors has been clearly documented in the context of plant defense [37–39], the biological relevance of such phenomenon is not clear. Whereas alerting a neighbor of an incoming threat may appear favorable if plants are genetically related, as postulated by the kin selection theory [40], this could be counterproductive in case the neighbor is a competitor for limited resources. Plants seldom grow in monocultures in nature and as the effect of egg-induced SAR diminishes with distance from the emitter plant, it is likely to alarm species other than the emitter. Therefore, alerting neighbors altruistically and regardless of kinship will strengthen the preexisting competition between species but may help to increase plant resistance on a community level in the field [41].

In conclusion, the question whether insects, plants, or both benefit from egg-triggered immune signaling in the dynamic interaction between plants, insects, and microbes is fascinating and not fully understood yet. Findings on how plants respond to EAMPs and how the consequential signaling events affect plant–herbivore–microbe interactions open the way for future investigations under more realistic natural conditions where plant communities are constantly challenged by multiple attackers.

## References

[1] Hilker M, Fatouros NE. Plant Responses to Insect Egg Deposition. Annu Rev Entomol. 2015;60:493–515. doi: 10.1146/annurev-ento-010814-020620 25341089

[2] Paniagua Voirol LR, Valsamakis G, Lortzing V, Weinhold A, Johnston PR, Fatouros NE, et al. Plant responses to insect eggs are not induced by egg-associated microbes, but by a secretion attached to the eggs. Plant Cell Environ. 2020;43:1815–26. doi: 10.1111/pce.13746 32096568

[3] Hundacker J, Bittner N, Weise C, Bröhan G, Varama M, Hilker M. Pine defense response to eggs of an herbivorous sawfly are elicited by an annexin-like protein. Plant Cell Environ. 2021;45:1033–48. doi: 10.1111/pce.14211 34713898

[4] Reymond P. The chemistry pf Plant-Insect Egg Interactions. Chimia (Aarau). 2022;76:914–21. doi: 10.2533/chimia.2022.914 38069786

[5] Gouhier-Darimont C, Schmiesing A, Bonnet C, Lassueur S, Reymond P. Signalling of *Arabidopsis thaliana* response to *Pieris brassicae* eggs shares similarities with PAMP-triggered immunity. J Exp Bot. 2013;64:665–74. doi: 10.1093/jxb/ers362 23264520 PMC3542055

[6] Wang C, Zhou M, Zhang X, Yao J, Zhang Y, Mou Z. A lectin receptor kinase as a potential sensor for extracellular nicotinamide adenine dinucleotide in *Arabidopsis thaliana*. Elife. 2017;6:e25474. doi: 10.7554/eLife.25474 28722654 PMC5560858

[7] Gouhier-Darimont C, Stahl E, Glauser G, Reymond P. The Arabidopsis lectin receptor kinase LecRK-I.8 Is involved in Insect Egg Perception. Front Plant Sci. 2019;10:623. doi: 10.3389/fpls.2019.00623 31134123 PMC6524003

[8] Stahl E, Brillatz T, Ferreira Queiroz E, Marcourt L, Schmiesing A, Hilfiker O, et al. Phosphatidylcholines from *Pieris brassicae* eggs activate an immune response in Arabidopsis. Elife. 2020;9:e60293. doi: 10.7554/eLife.60293 32985977 PMC7521926

[9] Groux R, Stahl E, Gouhier-Darimont C, Kerdaffrec E, Jimenez-Sandoval P, Santiago J, et al. Arabidopsis natural variation in insect egg-induced cell death reveals a role for LECTIN RECEPTOR KINASE-I.1. Plant Physiol. 2021;185:240–55. doi: 10.1093/plphys/kiaa022 33631806 PMC8133593

[10] Bassetti N, Caarls L, Bukovinszkine’Kiss G, El-Soda M, van Veen J, Bouwmeester K, et al. Genetic analysis reveals three novel QTLs underpinning a butterfly egg-induced hypersensitive response-like cell death in *Brassica rapa*. BMC Plant Biol. 2022;22:140. doi: 10.1186/s12870-022-03522-y 35331150 PMC8944062

[11] Stahl E, Hilfiker O, Reymond P. Plant-arthropod interactions: who is the winner? Plant J. 2018;93:703–28. doi: 10.1111/tpj.13773 29160609

[12] Alfonso E, Stahl E, Glauser G, Bellani E, Raaymakers TM, van den Ackerveken G, et al. Insect eggs trigger systemic acquired resistance against a fungal and an oomycete pathogen. New Phytol. 2021;232:2491–505. doi: 10.1111/nph.17732 34510462 PMC9292583

[13] Little D, Gouhier-Darimont C, Bruessow F, Reymond P. Oviposition by Pierid Butterflies Triggers Defense Responses in Arabidopsis. Plant Physiol. 2007;143:784–800. doi: 10.1104/pp.106.090837 17142483 PMC1803735

[14] Lortzing T, Kunze R, Steppuhn A, Hilker M, Lortzing V. Arabidopsis, tobacco, nightshade and elm take insect eggs as herbivore alarm and show similar transcriptomic alarm responses. Sci Rep. 2020;10:16281. doi: 10.1038/s41598-020-72955-y 33004864 PMC7530724

[15] Ojeda-Martinez D, Diaz I, Santamaria ME. Transcriptomic Landscape of Herbivore Oviposition in Arabidopsis: A Systematic Review. Front Plant Sci. 2022;12:772492. doi: 10.3389/fpls.2021.772492 35126411 PMC8815302

[16] Groux R, Fouillen L, Mongrand S, Reymond P. Sphingolipids are involved in insect egg-induced cell death in Arabidopsis. Plant Physiol. 2022;189:2535–53. doi: 10.1093/plphys/kiac242 35608326 PMC9342989

[17] Griese E, Caarls L, Bassetti N, Mohammadin S, Verbaarschot P, Bukovinszkine’Kiss G, et al. Insect egg-killing: a new front on the evolutionary arms-race between brassicaceous plants and pierid butterflies. New Phytol. 2021;230:341–53. doi: 10.1111/nph.17145 33305360 PMC7986918

[18] Hilfiker O, Groux R, Bruessow F, Kiefer K, Zeier J, Remond P. Insect eggs induce a systemic acquired resistance in Arabidopsis. Plant J. 2014;80:1085–94. doi: 10.1111/tpj.12707 25329965

[19] Fu ZQ, Dong X. Systemic acquired resistance: turning local infection into global defense. Annu Rev Plant Biol. 2013;64:839–63. doi: 10.1146/annurev-arplant-042811-105606 23373699

[20] Bigeard J, Colcombet J, Hirt H. Signaling mechanisms in pattern-triggered immunity (PTI). Mol Plant. 2015;8:521–39. doi: 10.1016/j.molp.2014.12.022 25744358

[21] Hartmann M, Zeier J. N-hydroxypipecolic acid and salicylic acid: a metabolic duo for systemic acquired resistance. Curr Opin Plant Biol. 2019;50:44–57. doi: 10.1016/j.pbi.2019.02.006 30927665

[22] Group Prime-A-Plant. Priming: Getting Ready for Battle. Mol Plant Microbe Interact. 2006;19:1062–71. doi: 10.1094/MPMI-19-1062 17022170

[23] Bernsdorff F, Döring AC, Gruner K, Schuck S, Bräutigam A, Zeier J. Pipecolic Acid Orchestrates Plant Systemic Acquired Resistance and Defense Priming via Salicylic Acid-Dependent and -Independent Pathways. Plant Cell. 2016;28:102–129. doi: 10.1105/tpc.15.00496 26672068 PMC4746677

[24] Stahl E, Bellwon P, Huber S, Schlaeppi K, Bernsdorff F, Vallat-Michel A, et al. Regulatory and Functional Aspects of Indolic Metabolism in Plant Systemic Acquired Resistance. Mol Plant. 2016;9:662–681. doi: 10.1016/j.molp.2016.01.005 26802249

[25] Orlovskis Z, Reymond P. *Pieris brassicae* eggs trigger interplant systemic acquired resistance against a foliar pathogen in Arabidopsis. New Phytol. 2020;228:1652–61. doi: 10.1111/nph.16788 32619278

[26] Caarls L, Pieterse CMJ, van Wees SCM. How salicylic acid takes transcriptional control over jasmonic acid signaling. Front Plant Sci. 2015;6:170. doi: 10.3389/fpls.2015.00170 25859250 PMC4373269

[27] Aerts N, Pereira Mendes M, van Wees SCM. Multiple levels of crosstalk in hormone networks regulating plant defense. Plant J. 2021;105:489–504. doi: 10.1111/tpj.15124 33617121 PMC7898868

[28] Bruessow F, Gouhier-Darimont C, Buchala A, Metraux JP, Reymond P. Insect eggs suppress plant defence against chewing herbivores. Plant J. 2010;62:876–85. doi: 10.1111/j.1365-313X.2010.04200.x 20230509

[29] Valsamakis G, Bittner N, Fatouros NE, Kunze R, Hilker M, Lortzing V. Priming by Timing: *Arabidopsis thaliana* Adjusts Its Priming Response to Lepidoptera Eggs to the Time of Larval Hatching. Front Plant Sci. 2020;11:619589. doi: 10.3389/fpls.2020.619589 33362842 PMC7755604

[30] Attaran E, Rostás W, Zeier J. Pseudomonas syringae Elicits Emission of the Terpenoid (E,E)-4,8,12-Trimethyl-1,3,7,11-Tridecatetraene in Arabidopsis Leaves Via Jasmonate Signaling and Expression of the Terpene Synthase TPS4. Mol Plant Microbe Interact. 2008;21:1482–97. doi: 10.1094/MPMI-21-11-1482 18842097

[31] Riedlmeier M, Ghirardo A, Wenig M, Knappe M, Knappe C, Koch K, et al. Monoterpenes Support Systemic Acquired Resistance within and between Plants. Plant Cell. 2017;29:1440–59. doi: 10.1105/tpc.16.00898 28536145 PMC5502447

[32] Shi X, Chen G, Tian L, Peng L, Xie W, Wu Q, et al. The Salicylic Acid-Mediated Release of Plant Volatiles Affects the Host Choice of *Bemesia tabaci*. 2016. Int J Mol Sci;17:1048. doi: 10.3390/ijms17071048 27376280 PMC4964424

[33] Huang J. Ultrastructure of Bacterial Penetration in Plants. Annu Rev Phytopathol. 1986;24:141–57. doi: 10.1146/annurev.py.24.090186.001041

[34] Katagiri F, Thilmony R, He SY. The Arabidopsis thaliana-pseudomonas syringae interaction. Arabidopsis Book. 2002;1:e0039. doi: 10.1199/tab.0039 22303207 PMC3243347

[35] Humphrey PT, Whiteman NK. Insect herbivory reshapes a native leaf microbiome. Nat Ecol Evol. 2020;4:221–29. doi: 10.1038/s41559-019-1085-x 31988447 PMC7332206

[36] Hou S, Tsuda K. Salicylic acid and jasmonic acid crosstalk in plant immunity. Essays Biochem. 2022;66:647–56. doi: 10.1042/EBC20210090 35698792

[37] Hu L. Integration of multiple volatile cues into plant defense responses. New Phytol. 2022;223:618–23. doi: 10.1111/nph.17724 34506634

[38] Vlot AC, Sales JH, Lenk M, Bauer K, Brambilla A, Sommer A, et al. Systemic propagation of immunity in plants. New Phytol. 2021;229:1234–50. doi: 10.1111/nph.16953 32978988

[39] Wang L, Erb M. Volatile uptake, transport, perception, and signaling shape a plant’s nose. Essays Biochem. 2022;66:695–702. doi: 10.1042/EBC20210092 36062590 PMC9528081

[40] Hamilton WD. The genetical evolution of social behaviour. I. J Theor Biol. 1964;7:1–16. doi: 10.1016/0022-5193(64)90038-4 5875341

[41] Pélissier R, Buendia L, Brousse A, Temple C, Ballini E, Fort F, et al. Plant neighbour-modulated susceptibility to pathogens in intraspecific mixtures. J Exp Bot. 2021;72:6570–80. doi: 10.1093/jxb/erab277 34125197 PMC8483782

